# The myokine meteorin‐like (metrnl) improves glucose tolerance in both skeletal muscle cells and mice by targeting AMPKα2

**DOI:** 10.1111/febs.15301

**Published:** 2020-04-19

**Authors:** Jung Ok Lee, Won Seok Byun, Min Ju Kang, Jeong Ah Han, Jiyoung Moon, Min‐Jeong Shin, Ho Jun Lee, Ji Hyung Chung, Jin‐Seok Lee, Chang‐Gue Son, Kwon‐Ho Song, Tae Woo Kim, Eun‐Soo Lee, Hong Min Kim, Choon Hee Chung, Kevin R. W. Ngoei, Naomi X. Y. Ling, Jonathan S. Oakhill, Sandra Galic, Lisa Murray‐Segal, Bruce E. Kemp, Kyoung Min Kim, Soo Lim, Hyeon Soo Kim

**Affiliations:** ^1^ Department of Anatomy Korea University College of Medicine Seoul Korea; ^2^ Department of Public Health Sciences Korea University Seoul Korea; ^3^ Department of Biotechnology CHA University Gyeonggi‐do Korea; ^4^ Liver and Immunology Research Center Oriental Medical College of Daejeon University Korea; ^5^ Department of Biomedical Science College of Medicine Korea University Seoul Korea; ^6^ Department of Biochemistry and Molecular Biology College of Medicine Korea University Seoul Korea; ^7^ Department of Internal Medicine Yonsei University College of Medicine Wonju Korea; ^8^ Protein Chemistry and Metabolism St Vincent's Institute of Medical Research University of Melbourne Fitzroy Vic. Australia; ^9^ Metabolic Signaling Laboratory St Vincent's Institute of Medical Research University of Melbourne Fitzroy Vic. Australia; ^10^ Mary MacKillop Institute for Health Research Australian Catholic University Fitzroy Vic. Australia; ^11^ Department of Internal Medicine Seoul National University College of Medicine and Seoul National University Bundang Hospital Seongnam Korea

**Keywords:** adipomyokine, AMPK, glucose uptake, Metrnl, type 2 diabetes

## Abstract

Meteorin‐like (metrnl) is a recently identified adipomyokine that beneficially affects glucose metabolism; however, its underlying mechanism of action is not completely understood. We here show that the level of metrnl increases *in vitro* under electrical pulse stimulation and *in vivo* in exercised mice, suggesting that metrnl is secreted during muscle contractions. In addition, metrnl increases glucose uptake via the calcium‐dependent AMPKα2 pathway in skeletal muscle cells and increases the phosphorylation of HDAC5, a transcriptional repressor of GLUT4, in an AMPKα2‐dependent manner. Phosphorylated HDAC5 interacts with 14‐3‐3 proteins and sequesters them in the cytoplasm, resulting in the activation of GLUT4 transcription. An intraperitoneal injection of recombinant metrnl improved glucose tolerance in mice with high‐fat‐diet‐induced obesity or type 2 diabetes, but not in AMPK β1β2 muscle‐specific null mice. Metrnl improves glucose metabolism via AMPKα2 and is a promising therapeutic candidate for glucose‐related diseases such as type 2 diabetes.

## Graphical abstract

We found that metrnl, known as an adipomyokine, is secreted during muscle contractions. Metrnl increases glucose uptake via AMPKα in skeletal muscle cells and increases the phosphorylation of HDAC5 and TBC1D1 in AMPKα‐dependent manner. Recombinant metrnl improves glucose tolerance in mice with obesity or type 2 diabetes. These results suggest that metrnl is a promising therapeutic candidate for diabetes.

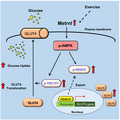

**Abbreviations**
ACC: acetyl‐CoA carboxylase
AMPK: AMP‐activated protein kinase
BAPTA‐AM: 1,2‐Bis (2‐aminophenoxy) ethane‐*N*,*N*,*N*′,*N*′‐tetraacetic acid tetrakis (acetoxymethyl ester
EPS: electrical pulse stimulation
GLUT4: glucose transporter type 4
GST: glutathione S‐transferase
GTT: glucose tolerance test
HDAC5: histone deacetylase 5
HFD: high‐fat diet
ICC: immunocytochemistry
MAPK: mitogen‐activated protein kinase
TBC1D1: TBC1 domain family member 1

## Introduction

Exercise has the potential to protect against metabolic disease, cardiovascular disease, cancer, and dementia [1]. In response to exercise, skeletal muscle cells secrete various proteins called myokines that elicit responses in an auto‐, para‐, or endocrine manner [2, 3]. Myokines are known to improve glucose homeostasis and insulin sensitivity [4], glucose tolerance [5], regulation of fat oxidation [6], and satellite cell proliferation [7, 8]. Adipose tissue is an endocrine organ that releases various adipokines to control systemic metabolism and energy homeostasis [9, 10]. Skeletal muscle contraction‐regulated myokines are secreted by adipocytes and are thus called adipomyokines; they are associated with beneficial, exercise‐induced metabolic effects [11, 12, 13]. We have previously analyzed the role of the adipomyokines irisin [14], fstl‐1 [15], resistin [16], and visfatin [17], which perform various functions in different organs.

Meteorin‐like hormone (metrnl), also known as cometin, subfatin, and IL‐39, is a secreted adipomyokine [18] expressed in various tissues, including the liver, heart, stromal cells, macrophages, spleen, and central nervous system [19, 20]. Metrnl is induced in skeletal muscles upon exercise and in white adipose tissue during exposure to cold [21, 22]. Metrnl stimulates energy expenditure and improves glucose tolerance and the expression of genes associated with thermogenesis in brown and beige adipocytes and anti‐inflammatory cytokines [22]. Furthermore, adipocyte‐specific knock‐out of metrnl exacerbates insulin resistance induced by a high‐fat diet (HFD), whereas adipocyte‐specific transgenic overexpression of metrnl prevents insulin resistance induced by HFD or leptin deletion, suggesting that adipocyte metrnl ameliorates overall insulin resistance by acting on local adipose tissue in an autocrine/paracrine fashion [23]. Until now, the expression and function of metrnl have been explored extensively in fat tissues, but few studies have considered the molecular mechanism of metrnl‐mediated antidiabetic effects in skeletal muscle.

AMP‐activated kinase (AMPK) is a master regulator of metabolic homeostasis and an energy‐sensing serine/threonine kinase [24]. During exercise, it is activated in skeletal muscles, adipose tissue, the liver, and other organs by events that increase the AMP/ATP ratio. Activated AMPK stimulates glucose uptake in skeletal muscle, induces fatty acid oxidation in adipose tissue, and reduces hepatic glucose production [25], indicating that AMPK plays a crucial role in the regulation of glucose homeostasis. Exercise is perhaps the most powerful physiological activator of AMPK.

In the present study, we determined whether exercise stimulates the expression of metrnl in skeletal muscle and investigated the effects of metrnl on glucose homeostasis using mouse models of obesity and diabetes. In addition, we investigated the molecular mechanisms responsible for improved glucose homeostasis in skeletal muscle cells and AMPK β1β2‐muscle‐specific null mice.

## Results

### Metrnl levels increased *in vivo* and *in vitro* muscle contraction models

**Fig. 1:**
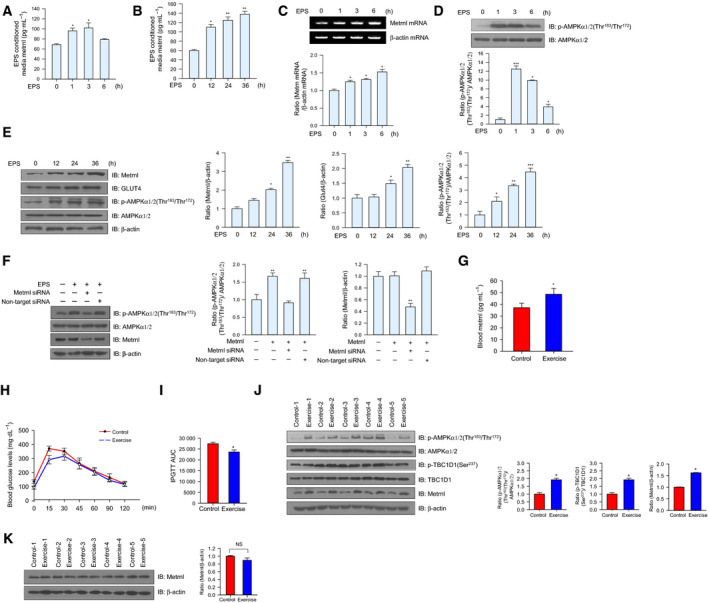
The level of metrnl increased *in vitro* and *in vivo* exercise models. (A, B) C2C12 myotubes were subjected to an acute or chronic electrical pulse stimulation (EPS), and the conditioned media (serum‐free DMEM) were analyzed using a metrnl ELISA kit. (C) Total mRNA was prepared from C2C12 myotubes after EPS, and RT‐PCR was performed using metrnl‐specific primers. PCR products were separated on a 1% agarose gel and visualized under ultraviolet light, with β‐actin as the positive control. (D) C2C12 myotubes were subjected to acute EPS. Lysates were analyzed by western blotting using anti‐phospho‐AMPKα1/2 (Thr^183^/Thr^172^) antibody, with AMPKα1/2 and β‐actin as the controls. (E) Total protein was prepared from C2C12 myotubes after chronic electric pulse stimulation, and western blot analysis was performed using metrnl, GLUT4, and phospho‐AMPKα1/2 (Thr^183^/Thr^172^) antibodies, with β‐actin and AMPKα1/2 as the controls. (F) C2C12 myoblasts were transiently transfected with metrnl siRNA for 24 h. Then, the cells were subjected on acute EPS. Cell lysates were analyzed by western blotting using anti‐phospho‐AMPKα (Thr^183^/Thr^172^), metrnl, AMPKα1/2 antibodies, with β‐actin as the controls. (G) BALB/C mice were divided into groups: sedentary (*n* = 10) and forced treadmill running (*n* = 10). Mice were sacrificed after chronic exercise, and the level of metrnl circulating in the blood was measured by ELISA. (H, I) Intraperitoneal (IP) GTT: blood glucose concentrations were measured after intraperitoneal administration of glucose (2 mg·kg^−1^ body weight). (J) Western blot analysis of phospho‐AMPKα1/2 (Thr^183^/Thr^172^), AMPKα1/2, phosphos‐TBC1D1 (Ser^237^), TBC1D1, and metrnl in thigh muscles of sedentary and exercise mice. β‐Actin is shown as a loading control. (K) Western blot analysis of metrnl in adipose tissues of sedentary and exercise mice. β‐Actin is shown as a loading control. Results are displayed as the mean ± SEM of five experiments. *P < 0.05, **P < 0.01, and ***P < 0.001 compared with control.

To verify whether metrnl was secreted or upregulated following muscle contractions, differentiated C2C12 myotube cells underwent electrical pulse stimulation (EPS) to mimic exercise. The concentration of metrnl increased in acute or chronic EPS‐conditioned media, implying that metrnl was secreted upon muscle stimulation (Fig. 1A,B), and the expression of metrnl mRNA also increased (Fig. 1C). In addition, the phosphorylation of AMPKα1/2, a key molecule in muscle contraction, increased in the cell lysate after acute or chronic EPS (Fig. 1D,E). To further understand the effect of metrnl on EPS‐induced AMPKα1/2 phosphorylation, we used siRNA‐mediated downregulation of metrnl to block the phosphorylation of AMPKα1/2 after acute EPS (Fig. 1F). In a chronic exercise mouse model (1 h·day^−1^ for 3 weeks), metrnl blood concentrations increased after forced treadmill running (Fig. 1G). Glucose tolerance was improved in chronic exercise mice (Fig. 1H,I). In addition to the expression of metrnl, the phosphorylation of AMPKα1/2 and TBC1D1 increased in the quadriceps femoris muscles of the chronic exercised mice (Fig. 1J). However, metrnl did not increase in adipocyte tissues (Fig. 1K), suggesting that the metrnl levels follow the plasma levels. Taken together, these results suggest that exercise increases the muscle contraction‐induced secretion of metrnl.

### Metrnl stimulated glucose uptake via AMPKα2 in skeletal muscle cells

**Fig. 2:**
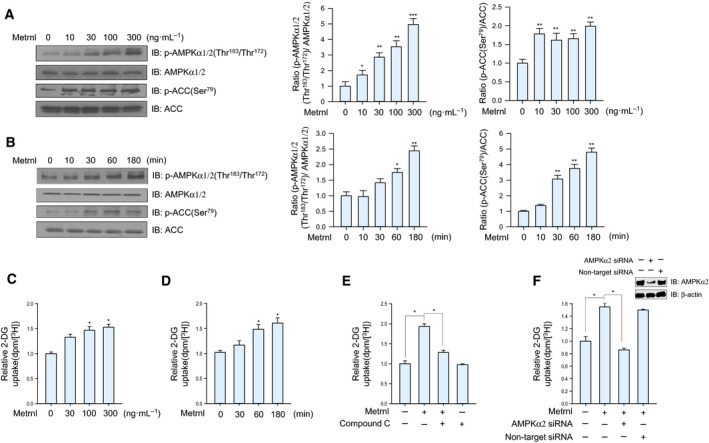
Metrnl stimulated glucose uptake via AMPK in skeletal muscle cells. (A) Dose‐dependent phosphorylation of AMPKα1/2 and ACC after metrnl treatment. C2C12 myoblasts were stimulated for 60 min at various metrnl concentrations. The cell lysates were analyzed by western blotting using antibodies against phospho‐AMPKα (Thr^183^/Thr^172^) and phospho‐ACC (Ser^79^), with AMPKα1/2 and ACC as the controls. (B) Time‐dependent phosphorylation of AMPKα1/2 and ACC after metrnl treatment. C2C12 cells were incubated with metrnl (100 ng·mL^−1^) for the indicated times. Cell lysates were analyzed by western blotting using antibodies against phospho‐AMPKα1/2 (Thr^183^/Thr^172^) and phospho‐ACC (Ser^79^), with AMPKα1/2 and ACC as the controls. (C) Dose‐dependent uptake of glucose with metrnl treatment. C2C12 myotubes were incubated with metrnl at several concentrations for 1 h and then assayed for glucose uptake. (D) Time‐dependent uptake of glucose with metrnl treatment. C2C12 myotubes were incubated with metrnl (100 ng·mL^−1^) for the indicated times and then assayed for glucose uptake. (E) C2C12 myotubes were treated with metrnl (100 ng·mL^−1^) for 1 h in the presence of compound C (10 µm) and then assayed for glucose uptake. (F) C2C12 myotubes were transiently transfected with AMPKα2 siRNA or non‐target siRNA, incubated with metrnl (100 ng·mL^−1^) for 1 h and then assayed for glucose uptake. Results are displayed as the mean ± SEM of five experiments. *P < 0.05, **P < 0.01, and ***P < 0.001.

To determine whether metrnl affects glucose homeostasis, we evaluated its effect on AMPKα1/2 phosphorylation in C2C12 mouse skeletal muscle cells. Metrnl treatment increased AMPKα1/2 phosphorylation in a dose‐ and time‐dependent manner (Fig. 2A,B) and also increased the phosphorylation of acetyl‐CoA carboxylase (ACC), a downstream substrate of AMPK. In addition, metrnl increased glucose uptake in dose ranges from 30 to 300 ng·mL^−1^ (Fig. 2C) and time points between 30 and 180 min in differentiated C2C12 myotubes (Fig. 2D). These effects were suppressed when AMPKα2 was inhibited by compound C or knocked down by siRNA (Fig. 2E,F), suggesting that metrnl stimulates glucose uptake via AMPKα2 in skeletal muscle cells.

### Metrnl increased AMPKα1/2 phosphorylation by increasing intracellular calcium concentrations

**Fig. 3:**
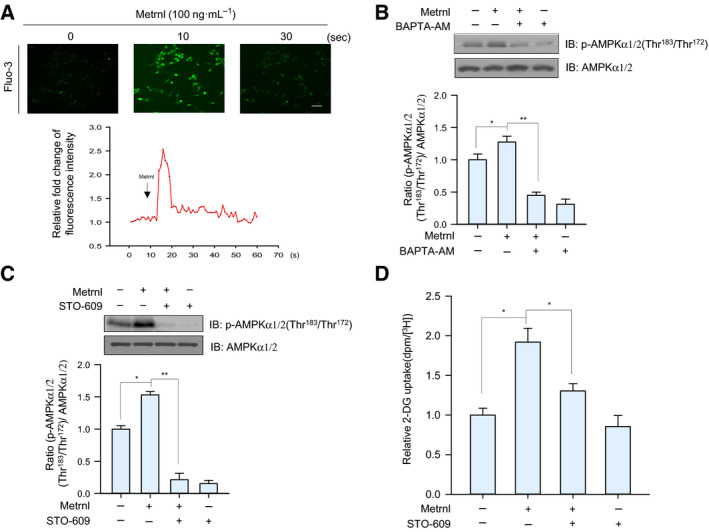
Metrnl activated AMPK by increasing intracellular calcium concentrations. (A) For Ca^2+^ detection, C2C12 myoblasts were pre‐incubated in Fluo‐3 AM (10 µm) for 30 min. The Ca^2+^ response was measured after C2C12 incubated with metrnl (100 ng·mL^−1^). The Ca^2+^ concentration correlates with the fluorescence intensity. Scale bars, 100 μm (*n* = 5). (B) C2C12 myoblasts were pre‐treated with the membrane‐impermeable calcium chelator BAPTA‐AM (5 μm) and then incubated with metrnl (100 ng·mL^−1^) for 60 min. Cell lysates were analyzed by western blotting using anti‐phospho‐AMPKα1/2 (Thr^183^/Thr^172^) antibody, with AMPKα1/2 as the control. (C) C2C12 myoblasts were pre‐treated with the CaMKK2 inhibitor STO‐609 (5 µm) and then treated with metrnl (100 ng·mL^−1^). Cell lysates were analyzed by western blotting using anti‐phospho‐AMPKα1/2 (Thr^183^/Thr^172^) antibody, with AMPKα1/2 as the control. (D) C2C12 myotubes were treated with metrnl (100 ng·mL^−1^) for 1 h in the presence of STO‐609 (5 µm) and then assayed for glucose uptake. Results are displayed as the mean ± SEM of five experiments. *P < 0.05 and **P < 0.01.

Glucose uptake can be regulated by calcium‐sensitive contraction‐dependent mechanisms [26]; thus, we hypothesized that calcium could be involved in metrnl‐mediated AMPKα1/2 activation. Metrnl increased the fluorescence intensity of cells stained with Fluo‐3 AM, a calcium dye (Fig. 3A), whereas pre‐treatment with BAPTA‐AM, an intracellular calcium chelator, blocked metrnl‐induced AMPKα1/2 phosphorylation (Fig. 3B). Moreover, when CaMKK2 (calcium/calmodulin‐dependent protein kinase 2, upstream of AMPK) was inhibited using STO‐609, metrnl‐induced AMPKα1/2 phosphorylation and glucose uptake were blocked (Fig. 3C,D). These results suggest that metrnl stimulates glucose uptake via calcium‐mediated AMPKα phosphorylation.

### Metrnl increased glucose uptake via p38 MAPK pathway

**Fig. 4:**
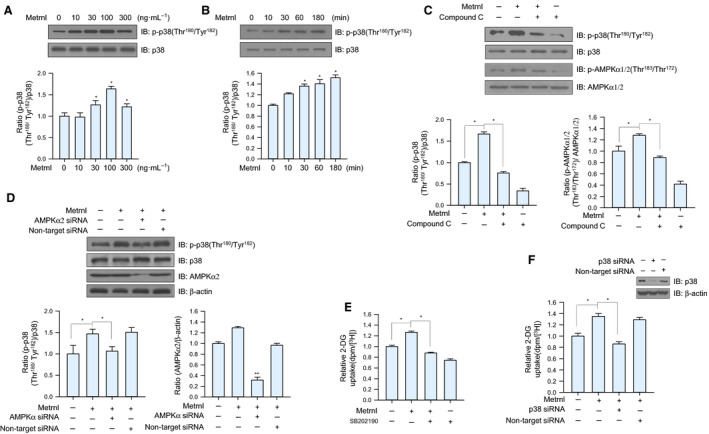
Metrnl increased glucose uptake via the p38 MAPK pathway. (A) C2C12 myoblasts were stimulated for 60 min with several concentrations of metrnl. The cell lysates were analyzed by western blotting using anti‐phospho‐p38 MAPK antibody, with p38 MAPK as the control. (B) Time‐dependent phosphorylation of p38 MAPK after metrnl treatment. C2C12 myoblasts were incubated with metrnl (100 ng·mL^−1^) for the indicated times. Cell lysates were analyzed by western blotting using anti‐phospho‐p38 MAPK antibody, with p38 MAPK as the control. (C) C2C12 myoblasts were pre‐treated with compound C (10 μm), then treated with metrnl (100 ng·mL^−1^). Cell lysates were analyzed by western blotting using antibodies against phospho‐p38 MAPK and phospho‐AMPKα1/2(Thr^183^/Thr^172^), with p38 MAPK and AMPKα1/2 as the controls. (D) C2C12 myoblasts were transiently transfected with AMPKα2 siRNA or non‐target siRNA. Cell lysates were analyzed by western blotting using anti‐phospho‐p38 MAPK antibody, with p38, AMPKα2, and β‐actin as the controls. (E) C2C12 myotubes were treated with metrnl (100 ng·mL^−1^) for 1 h in the presence of SB202190 (20 µm) and then assayed for glucose uptake. (F) C2C12 myotubes were transiently transfected with p38 MAPK siRNA or non‐target siRNA, incubated with metrnl (100 ng·mL^−1^) for 1 h, and then assayed for glucose uptake. Results are displayed as the mean ± SEM of five experiments. *P < 0.05 and **P < 0.01.

The activation of p38 mitogen‐activated protein kinase (MAPK) increases glucose uptake via enhanced GLUT4 translocation in cardiomyocytes [27, 28]. To assess the effect of metrnl on p38 MAPK in skeletal muscle cells, we measured p38 MAPK phosphorylation in C2C12 myoblasts after metrnl treatment. Metrnl increased p38 MAPK phosphorylation in a dose‐ and time‐dependent manner (Fig. 4A,B). We then investigated the role of AMPKα2 in the metrnl‐mediated phosphorylation of p38 MAPK and found that inhibition or knockdown of AMPK suppressed the metrnl‐mediated phosphorylation of p38 MAPK (Fig. 4C,D). To confirm these findings, we examined the effect of p38 MAPK inhibition on glucose uptake. Metrnl‐induced glucose uptake was suppressed when p38 MAPK was inhibited by SB202190 or siRNA knockdown (Fig. 4E,F). These results demonstrate that p38 MAPK is involved in metrnl‐mediated glucose uptake as a downstream of AMPK.

### Metrnl regulated the binding of HDAC5 to the GLUT4 promoter

**Fig. 5:**
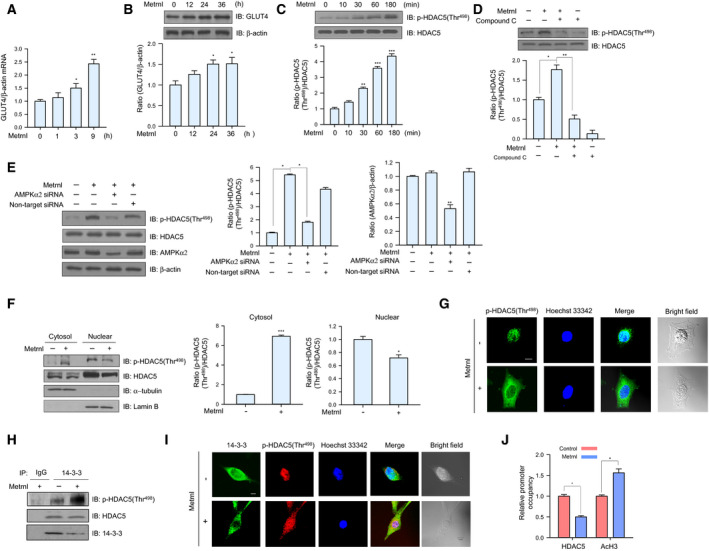
Metrnl increased GLUT4 expression by stimulating HDAC5 phosphorylation. (A) Total mRNA from C2C12 myoblasts was prepared after metrnl (100 ng·mL^−1^) treatment for the indicated times, and real‐time qRT‐PCR was performed using GLUT4‐specific primers, with β‐actin mRNA as the positive control. (B) C2C12 myoblasts were treated with metrnl (100 ng·mL^−1^) for the indicated times. The cell lysates were analyzed by western blotting using anti‐GLUT4 antibody, with β‐actin as the control. (C) Time‐dependent phosphorylation of HDAC5 after metrnl treatment. C2C12 myoblasts were incubated with metrnl (100 ng·mL^−1^) for the indicated times. Cell lysates were analyzed by western blotting using anti‐phospho‐HDAC5 (Thr^498^) antibody, with HDAC5 as the control. (D) C2C12 myoblasts were pre‐treated with compound C (10 μm) and then treated with metrnl (100 ng·mL^−1^). Cell lysates were analyzed by western blotting using anti‐phospho‐HDAC5 (Thr^498^) antibody, with HDAC5 as the control. (E) C2C12 myoblasts were transiently transfected with AMPKα2 siRNA or non‐target siRNA. Cell lysates were analyzed by western blotting using antibodies against phospho‐HDAC5 (Thr^498^), AMPKα2, and HDAC5, with β‐actin as the controls. (F) C2C12 myoblasts were treated with metrnl (100 ng·mL^−1^). Cytosolic and nuclear proteins were extracted from the cells. HDAC5 phosphorylation was evaluated by western blot analysis, with HDAC5 as the control. Western blotting was performed on nuclear and cytosolic fractions to detect nuclear (lamin B) and cytosolic (α‐tubulin) marker proteins. (G) Representative images of phospho‐HDAC5 treated with metrnl for 30 min. Scale bars, 10 μm (*n* = 5). (H) C2C12 myoblasts were immunoprecipitated with anti‐14‐3‐3 antibody, followed by western blotting using anti‐phospho‐HDAC5, HDAC5, and 14‐3‐3 antibodies. (I) Representative images (phospho‐HDAC5 and 14‐3‐3 objective images) of cells treated with metrnl for 1 h. Scale bars, 10 μm (*n* = 5). (J) The relative occupancy of HDAC5 and AcH3 on the GLUT4 promoter was assessed using a ChIP analysis following 60 min of metrnl (100 ng·mL^−1^) treatment. The ChIP data represent the ratio of IP values for each region relative to the input. The results shown are from three independent experiments. Other results are displayed as the mean ± SEM of five experiments. *P < 0.05, **P < 0.01, and ***P < 0.001.

Histone deacetylase 5 (HDAC5) is a corepressor of GLUT4 transcription that is exported from the nucleus after phosphorylation [29], and the chaperone protein 14‐3‐3 is known to mediate the nuclear export of HDAC5 [30]. We found that metrnl increased the mRNA and protein expression of GLUT4 (Fig. 5A,B) and increased HDAC5 phosphorylation in a time‐dependent manner (Fig. 5C). The phosphorylation of HDAC5 was not observed following the inhibition or knockdown of AMPKα2 (Fig. 5D,E), suggesting that metrnl increases the phosphorylation of HDAC5 via AMPKα2. We used cytosolic fractionation and immunocytochemistry (ICC) to confirm that metrnl induced the cytosolic translocation of phosphorylated HDAC5 (Fig. 5F,G). In immunoprecipitation (IP) and ICC experiments, metrnl also increased the interaction between phosphorylated HDAC5 and 14‐3‐3 (Fig. 5H,I), suggesting that 14‐3‐3 helps to sequester HDAC5 in the cytoplasm. To confirm whether metrnl affects HDAC5 binding to the GLUT4 promoter, we performed chromatin immunoprecipitation (ChIP) assays. Notably, metrnl treatment reduced HDAC5 binding to the GLUT4 promoter region and increased histone H3 acetylation in the same region (Fig. 5J). These data suggest that metrnl could upregulate GLUT4 expression by regulating the binding of HDAC5 to the GLUT4 promoter.

### Metrnl stimulated translocation of GLUT4 via TBC1D1 phosphorylation

**Fig. 6:**
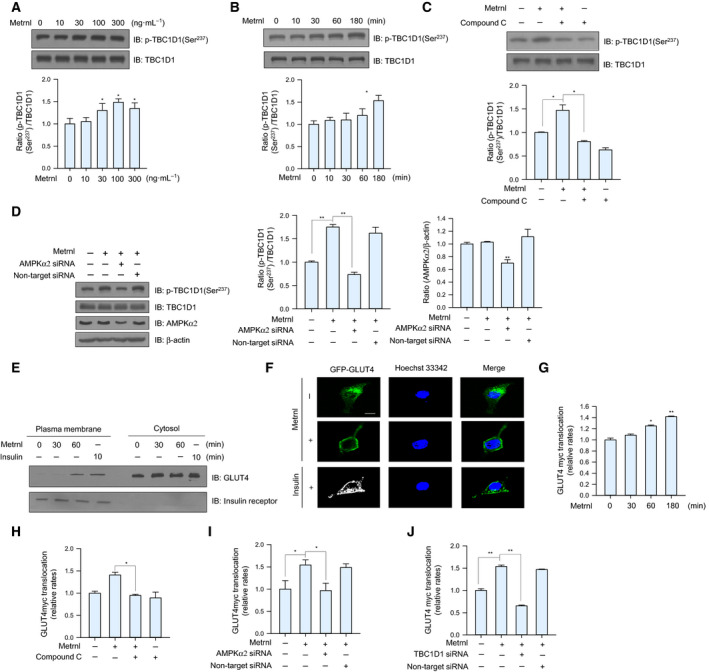
Metrnl stimulated GLUT4 translocation by AMPK‐induced TBC1D1 phosphorylation. (A) C2C12 myoblasts were stimulated for 1 h with different concentrations of metrnl. Cell lysates were analyzed by western blotting using anti‐phospho‐TBC1D1 (Ser^237^) antibody, with TBC1D1 as the control. (B) C2C12 myoblasts were incubated with metrnl (100 ng·mL^−1^) for the indicated times. Cell lysates were analyzed by western blotting using anti‐phospho‐TBC1D1 (Ser^237^) antibody, with TBC1D1 as the control. (C) C2C12 myoblasts were pre‐treated with compound C (10 μm) and then treated with metrnl (100 ng·mL^−1^). Cell lysates were analyzed by western blotting using anti‐phospho‐TBC1D1 (Ser^237^) antibody, with TBC1D1 as the control. (D) C2C12 myoblasts were transiently transfected with AMPKα2 siRNA or non‐target siRNA. Cell lysates were analyzed by western blotting using anti‐phospho‐TBC1D1 (Ser^237^), AMPKα2, TBC1D1 antibodies, with β‐actin as the controls. (E) C2C12 myoblasts treated with metrnl (100 ng·mL^−1^) or insulin (100 nm) were lysed and then fractionated into the plasma membrane and cytosol. Plasma membrane (PM) and cytosol proteins were analyzed by western blotting using anti‐GLUT4 antibody, with insulin receptor (IR) as a plasma membrane marker. (F) Representative images (GLUT4, Hoechst, and merged) of cells treated with metrnl for 1 h. Insulin (100 nm) was used as the positive control. Scale bars, 10 μm (*n* = 5). (G) Surface expression of GLUT4myc with metrnl treatment. L6‐GLUT4myc myotubes were incubated with metrnl at several time points for 3 h, and then, cell surface expression of GLUT4myc was detected using an antibody‐coupled colorimetric absorbance assay. (H) L6‐GLUT4myc myotubes were treated with metrnl (100 ng·mL^−1^) for 1 h in the presence of compound C (10 µm), and then, cell surface expression of GLUT4myc was detected using an antibody‐coupled colorimetric absorbance assay. (I, J) L6‐GLUT4myc myotubes were transiently transfected with AMPKα2 or TBC1D1 siRNA for 48 h before metrnl (100 ng·mL^−1^) treatment for 1 h. The cell surface expression of GLUT4myc was detected using an antibody‐coupled colorimetric absorbance assay. Results are displayed as the mean ± SEM of five experiments. *P < 0.05 and **P < 0.01.

TBC1D1 is a Rab‐GTPase‐activating protein involved in GLUT4 trafficking and is known to be activated by AMPK [27, 28]. We therefore tested whether TBC1D1 was involved in metrnl‐mediated glucose regulation. Metrnl induced TBC1D1 (Ser^237^) phosphorylation in a dose‐ and time‐dependent manner (Fig. 6A,B), but those effects were not observed when AMPKα2 was inhibited or knocked down (Fig. 6C,D). To confirm that result, we performed membrane fractionation and ICC experiments and found that metrnl increased GLUT4 translocation to the plasma membrane (Fig. 6E,F). Insulin was used as positive control for GLUT4 translocation. The cell surface localization of GLUT4myc was also measured using a colorimetric assay, which showed that metrnl increased plasma membrane GLUT4myc in a time‐dependent manner (Fig. 6G), which was not observed when AMPKα2 was inhibited or knockdown (Fig. 6H,I). In addition, knockdown with TBC1D1 siRNA suppressed the metrnl‐induced translocation of GLUT4 to the plasma membrane (Fig. 6J). These results suggest that metrnl stimulates translocation of GLUT4 via AMPKα‐mediated TBC1D1 phosphorylation.

### Metrnl stimulated AMPKα1/2 phosphorylation and glucose uptake in mouse primary myoblast cells

**Fig. 7:**
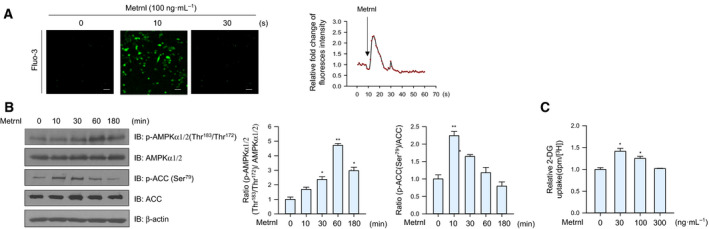
Metrnl regulated AMPK phosphorylation and glucose uptake in mouse primary myoblast cells. (A) For Ca^2+^ detection, myoblasts were pre‐incubated with Fluo‐3 AM (5 μm) for 30 min. After metrnl treatment, the Ca^2+^ response was measured using a confocal microscope. Scale bars, 100 μm (*n* = 5). (B) Mouse primary myoblast cells were stimulated with metrnl for the indicated times. Cell lysates were analyzed by western blotting using antibodies against phospho‐AMPKα1/2(Thr^183^/Thr^172^) and phospho‐ACC (Ser^79^), with AMPKα1/2, ACC, and β‐actin as the controls. (C) Dose‐dependent glucose uptake with metrnl treatment. Primary myoblasts were differentiated into myotubes, incubated with metrnl at several concentrations for 1 h, and then assayed for glucose uptake. Results are displayed as the mean ± SEM of five experiments. *P < 0.05 and **P < 0.01.

To assess the physiological relevance of metrnl, we investigated its effects in primary myoblasts prepared from the quadriceps femoris tissue of wild‐type (WT) mice (BALB/c). Metrnl significantly increased the calcium levels of primary myoblasts, with maximum fluorescence detected after 10 s (Fig. 7A). In addition, metrnl increased the phosphorylation of AMPKα1/2 and its downstream target ACC in a time‐dependent manner (Fig. 7B) and increased glucose uptake in differentiated primary myotubes (Fig. 7C), further supporting the biological relevance of metrnl.

### Metrnl administration improved glucose tolerance in animal models

**Fig. 8:**
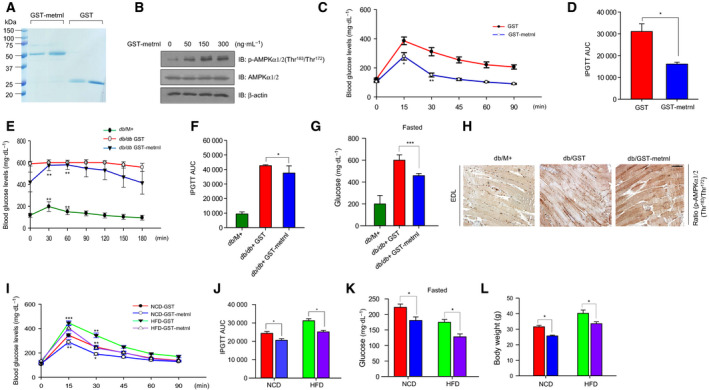
Metrnl improved glucose tolerance in mouse models. (A) Recombinant GST‐metrnl and GST proteins were isolated using glutathione beads. The beads were washed three times with washing buffer, eluted, and analyzed by SDS/PAGE and subsequent Coomassie staining. (B) C2C12 cells were treated with recombinant GST‐metrnl. Cell lysates were analyzed with western blotting using anti‐phospho‐AMPKα1/2(Thr^183^/Thr^172^) antibody, with AMPKα1/2 and β‐actin as the controls. (C, D) Blood glucose concentrations and area under the curve (AUC) results for the glucose tolerance test (GTT) in C57BL/6 mice injected with recombinant GST‐metrnl or GST proteins. (E, F) Blood glucose concentrations and AUC results for the GTT in db/M^+^, db/db + GST, and db/db + GST‐metrnl mice. (G) Fasting glucose levels results for the GTT in db/M^+^, db/db + GST, and db/db + GST‐metrnl mice. The mice fasted for 12 h, and tail vein blood was used to measure in the blood glucose levels. (H) Representative images of immunohistochemical detection of p‐AMPKα1/2 (Thr^183^/Thr^172^) in the extensor digitorum longus (EDL) muscles of db/M^+^, db/db + GST, and db/db + GST‐metrnl mice (scale bar = 100 μm). (I, J) Blood glucose concentrations and AUC results for the GTT in mice fed an HFD or NCD in NCD‐GST, NCD‐GST‐metrnl, HFD‐GST, and HFD‐GST‐metrnl. (K) Fasting glucose levels in mice fed an HFD or NCD in NCD‐GST, NCD‐GST‐metrnl, HFD‐GST, and HFD‐GST‐metrnl. The mice fasted for 12 h and tail vein blood was used to measure in the blood glucose levels. (L) Body weight of high‐fat‐diet‐induced obesity C57BL/6 mice. Groups were compared using analysis of variance (ANOVA) with Duncan's multiple range test. Results are displayed as the mean ± SEM of five experiments. *P < 0.05, **P < 0.01, and ***P < 0.001.

To investigate the effect of metrnl on glucose tolerance *in vivo*, we prepared recombinant GST‐tagged metrnl proteins and GST proteins using *Escherichia coli* (Fig. 8A). GST‐metrnl treatment increased AMPKα1/2 phosphorylation in C2C12 cells (Fig. 8B), confirming the biological activity of the recombinant protein. We then administered the metrnl‐GST to C57BL/6 mice (*n* = 12 per group) by intraperitoneal injection. GST‐metrnl reduced blood glucose levels and improved glucose tolerance (Fig. 8C,D). To confirm the effect of metrnl in a disease model, we administered GST‐metrnl to type 2 diabetic (db/db) mice and found that it ameliorated their impaired glucose tolerance (Fig. 8E,F). In addition, GST‐metrnl lowered glucose level in the blood and increased AMPKα1/2 phosphorylation in the extensor digitorum longus (EDL) muscles of db/M^+^, db/db + GST, and db/db + GST‐metrnl mice (Fig. 8G,H). To assess the chronic effect of metrnl on glucose tolerance in HFD‐induced obese mice, GST‐metrnl was intraperitoneally administered (at 48‐h intervals) for 8 weeks and significantly improved glucose tolerance in both the normal chow diet (NCD) and HFD groups (Fig. 8I,J). GST‐metrnl also lowered glucose levels and body weigh in HFD mice (Fig. 8K,L). Taken together, these results demonstrate that metrnl improves glucose tolerance in animal models.

### Metrnl did not improve glucose tolerance in AMPK β1β2 muscle‐specific null mice

**Fig. 9:**
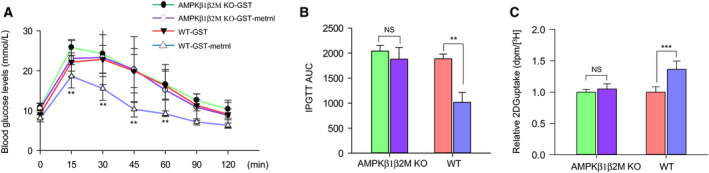
Metrnl did not improve glucose tolerance in AMPK β1β2M‐KO mice. (A, B) Blood glucose concentrations and area under the curve (AUC) results for the glucose tolerance test (GTT) in AMPK β1β2M‐KO mice injected with recombinant GST‐metrnl or GST proteins. (C) Extensor digitorum longus (EDL) tissues isolated from AMPK WT and AMPK β1β2M‐KO mice were incubated with GST‐metrnl or GST (4 µg·mL^−1^), and then, the uptake of 2‐deoxyglucose (2‐DG) was measured. The data are presented as the mean relative 2‐DG uptake (dpm/[H^3^] ± SD) based on 12–13 mice per group. **P < 0.01 and ***P < 0.001.

The β1 and β2 subunits of AMPK are required for the assembly of AMPK heterotrimers and are important for regulating enzyme activity. Mice lacking both the β1 and β2 isoforms in their skeletal muscle (β1β2M‐KO) have a drastically lower capacity for treadmill running and contraction‐stimulated glucose uptake [31]. To confirm the role of metrnl on the AMPK‐mediated glucose metabolism, we administered recombinant GST‐metrnl into AMPK β1β2M‐KO mice. The intraperitoneal injection of GST‐metrnl improved glucose tolerance in WT mice, but not in AMPK β1β2M‐KO mice (Fig. 9A,B). To further characterize the role of AMPK *in vivo*, we isolated the EDL muscles of WT and AMPK β1β2M‐KO mice and measured their glucose uptake ability. Metrnl‐GST increased glucose uptake in the EDL muscles of WT mice, but not those of AMPK β1β2M‐KO mice (Fig. 9C). These results clearly demonstrate that metrnl improves glucose tolerance via AMPK *in vivo*.

## Discussion

Exercise‐induced myokines are recognized as central players in both the prevention and treatment of type 2 diabetes. To date, several hundred myokines in the muscle secretome have been identified, a subpopulation of which are specifically induced by skeletal muscle contractions [32]. However, the bioactivity of many of these myokines and the mechanisms through which they act have either not yet been characterized or remain poorly understood. Among those many myokines, it is well known that metrnl is induced upon exercise [21, 22] and has antidiabetic effects by enhancing the browning of white adipose tissue or activating adipocyte differentiation [22, 23], suggesting that metrnl has therapeutic potential for metabolic diseases. However, the underlying molecular mechanisms by which metrnl improves glucose homeostasis have not been fully explained.

We observed that acute and chronic EPS increased the secretion and expression of metrnl into conditioning media and cell lysates and the phosphorylation of AMPKα1/2 in C2C12 myotubes (Fig. 1A–E). In addition, we investigated how acute and chronic exercise affected metrnl expression and glucose tolerance. Mice exposed to chronic exercise showed improved glucose tolerance compared with controls (Fig. 1H,I). Interestingly, the concentration of metrnl in the blood did not increase following acute exercise (data not shown), but it did increase in the mice exposed to chronic exercise (Fig. 1G), indicating that only chronic endurance training can induce metrnl‐mediated antidiabetic effects. However, metrnl mRNA expression increased in the acute EPS system (Fig. 1C). Therefore, the acute exercise model did not completely exclude the effect of increased metrnl levels. According to the previous reports, acute exercise enhances glucose homeostasis by activating insulin signaling and GLUT4 translocation [33, 34]. On the other hand, some human and animal studies have reported that acute exercise does not induce insulin signaling in skeletal muscles [35, 36] or metrnl mRNA expression [37]. Those reports support our results. Moderate endurance exercise seems to acutely increase insulin signaling [38], whereas short or light resistance and endurance training show no effect [33]. In addition, the time point after exercise at which the effect of exercise is studied appears to be highly relevant. A recent review from Frig and Richter found that exercise‐induced increases in glucose uptake occurred during a critical time point of 3–4 h after exercise, indicating a time‐dependent course in the activation of exercise‐induced molecular signaling [39] that could explain why the metrnl level in the blood of our acute exercise models did not increase: In our acute exercise experiment, we performed the glucose tolerance test (GTT) 1 h after exercise. To fully determine whether acute exercise increases metrnl levels in the blood, a further study using a different acute exercise protocol is needed.

Exercise increases glucose homeostasis by activating AMPK through an AMP‐dependent pathway or a Ca^2+^‐dependent pathway. To investigate the mechanism of metrnl‐mediated AMPK activation, we measured intracellular Ca^+2^ concentrations upon metrnl stimulation. In C2C12 myotubes and primary muscle cells, metrnl induced an increase in the calcium ion concentration (Figs 3A and 7A) and an increase in AMPKα1/2 phosphorylation (Figs 3B and 7B). It is well known that AMPKα2 can be directly phosphorylated at Thr172 by the calcium‐sensitive kinase CAMKK2 in response to calcium flux [40], which thus links calcium signaling to the regulation of energy metabolism by AMPK [41]. We observed that metrnl mediated AMPK phosphorylation and that glucose uptake was inhibited by STO‐609, a CAMMK2 inhibitor (Fig. 3C,D). Therefore, increased intracellular calcium caused by metrnl activates CAMKK2 and leads to both the activation of AMPK activity and an increase in glucose uptake.

AMPK increases GLUT4 transcription by phosphorylating HDAC5 [42]. Therefore, we examined whether metrnl‐mediated AMPKα1/2 phosphorylation increased GLUT4 expression by regulating HDAC5. Firstly, we found that metrnl increased the phosphorylation of HDAC5 (Fig. 5C) and the interaction between 14‐3‐3 and HDAC5 (Fig. 5H). AMPKα2 inhibition blocked metrnl‐mediated HDAC5 phosphorylation (Fig. 5D,E), implying that metrnl increases GLUT4 transcription via an AMPK‐HDAC5 signaling pathway. HDAC5 inhibitors are a novel treatment for diabetes mellitus that increases GLUT4 gene expression [43, 44], suggesting that metrnl might also treat diabetes by increasing GLUT4 expression.

Glucose uptake is promoted through a signaling cascade that contains several spatially distinct phosphorylation events that together move glucose transporters (GLUT4) to the plasma membrane, which upregulates glucose transport into the cell [45]. Specifically, AMPK mediates glucose uptake through TBC1D1 and p38 MAPK phosphorylation [27, 28, 46, 47]. TBC1D1 contains an AMPKα1/2 phosphorylation site (Ser237) [48], and TBC1D4 (AS160) phosphorylation (Thr642) is required for insulin‐dependent glucose uptake in skeletal muscle cells [45]. In this study, metrnl increased TBC1D1 phosphorylation (Ser237) (Fig. 6A,B) but did not alter TBC1D4 phosphorylation (Thr642) (data not shown), suggesting that metrnl stimulates GLUT4 translocation via the AMPK‐TBC1D1 axis separate from insulin signaling. In addition, metrnl induced the phosphorylation of p38 MAPK in a dose‐ and time‐dependent manner (Fig. 4A,B), and glucose uptake was decreased by inhibiting p38 MAPK (Fig. 4E,F), implying that metrnl enhances glucose uptake through an AMPK‐p38MAPK pathway. Taken together, our results show that metrnl regulates glucose homeostasis by separately activating TBC1D1 and p38 MAPK signaling pathways. To summarize our *in vitro* results, metrnl stimulates glucose uptake through Ca^2+^‐CAMKK2‐AMPK‐HDAC5‐GLUT4‐p38‐TBC1D1‐dependent signaling.

In this study, we found that metrnl improves glucose tolerance (Fig. 8C,D) and decreases the concentration of glucose in the blood in mouse models of obesity and type 2 diabetes (Fig. 8G,K). In addition, in the db/db mouse model, the phosphorylation of AMPKα1/2 in the EDL muscles increased after metrnl treatment (Fig. 8H). The body weight also decreased after metrnl treatment in the HFD mice (Fig. 8L). These data demonstrate that metrnl has an antidiabetic effect in mouse models of diabetes and HFD‐induced obesity. To confirm the role of AMPK in the antidiabetes effects of metrnl, we tested how metrnl affected glucose tolerance in AMPK β1β2M‐KO mice. Metrnl did not improve glucose tolerance or uptake in AMPK β1β2M‐KO mice, but it improved both in WT mice (Fig. 9A–C), demonstrating that metrnl improves glucose tolerance *in vivo* via the AMPK signaling pathway.

Interestingly, recent reports by Jung *et al*. and Bae *et al*. [49, 50] strongly support our data by showing that metrnl alleviates inflammation and improves insulin resistance through AMPK or PPARδ‐dependent signaling in skeletal muscles. Our results differ from those previous results in important ways. First, they investigated the effect of metrnl on glucose regulation only in HFD‐fed obese mice. In contrast, we examined the function of metrnl on glucose regulation using mouse models of both obesity and diabetes and demonstrated that AMPK is a key player in metrnl's antidiabetic effects using AMPK β1β2M‐KO mice. Second, we characterized the mechanism of metrnl‐mediated glucose homeostasis in skeletal muscle cells.

In conclusion, we have shown that metrnl has antidiabetic effects via a Ca^2+^‐CAMKK2‐AMPK‐HDAC5‐GLUT4‐p38‐TBC1D1 signaling pathway. Taken together, our results demonstrate that metrnl is an attractive therapeutic target for treating for metabolic syndrome.

## Materials and methods

### Reagents

Antibodies against AMPKα1/2, AMPKα2, p‐AMPKα1/2(Thr^183^/Thr^172^), phospho‐ACC (Ser^79^), ACC, and 14‐3‐3 were obtained from Abcam (San Francisco, CA, USA). TBC1D1 and p‐TBC1D1 (Ser^237^) were purchased from Merck Millipore (Darmstadt, Germany). Antibodies against HDAC5 and p38 MAPK were purchased from Cell Signaling Technology (Danvers, MA, USA). Antibodies against β‐actin were purchased from Sigma‐Aldrich (St. Louis, MO, USA). Antibodies against phospho‐p38 (Thr^180^/Tyr^182^), α‐tubulin, GLUT4, and lamin B were purchased from Santa Cruz Biotechnology (Santa Cruz, CA, USA). Phospho‐HDAC5 (Thr^498^) antibodies were from Thermo Fisher Scientific (Rockford, IL, USA). Horseradish peroxidase (HRP)‐conjugated goat anti‐rabbit IgG and goat anti‐mouse secondary antibodies were purchased from Enzo Life Sciences (Farmingdale, NY, USA). Metrnl was obtained from Cusabio (Wuhan, Hubei, China), and 1, 2‐bis (*o*‐aminophenoxy) ethane‐*N*, *N*, *N*′, *N*′‐tetraacetic acid (BAPTA)‐AM was purchased from Abcam. Compound C and STO‐609 were obtained from Calbiochem (San Diego, CA, USA). Protein A‐agarose beads were obtained from GE Healthcare (Piscataway, NJ, USA). The fluorescent Ca^2+^ indicator Fluo‐3 AM and Hoechst 33342 were obtained from Invitrogen (Leiden, the Netherlands).

### C2C12 myoblast cell culture and differentiation of C2C12 myoblasts

C2C12 myoblasts [American Type Culture Collection (ATCC), Manassoas, VA, USA] were cultured at 37 °C in 5% CO_2_ in Dulbecco's modified Eagle's medium (DMEM) supplemented with 10% FBS and 1% antibiotics. We induced skeletal muscle differentiation at 80–90% confluence of the myoblasts by changing the growth medium to differentiation medium (DMEM + 2% horse serum). After 5 days, the myotubes were used.

### Ca^2+^ measurement

Cells were treated with 5 μm Fluo‐3 AM in regular culture medium at 37 °C for 30 min, then washed, and incubated for 15 min in regular medium (without Fluo‐3 AM) to complete the de‐esterification process. Cells were treated with metrnl, and the culture plates were placed on a temperature‐controlled confocal microscope (Zeiss LSM700 Meta; Zeiss, Oberkochen, Germany) at 200× magnification. The excitation and emission wavelengths for signal detection were 488 and 515 nm, respectively.

### Immunoblot analyses

Following various experimental manipulations, the culture medium was removed, the cells were washed twice with ice‐cold PBS and then lysed with 70 μL of lysis buffer [50 mm Tris/HCl (pH 7.4), 1% Triton X‐100, 0.25% sodium deoxycholate, 150 mm EDTA, 1 mm sodium orthovanadate (Na_3_VO_4_), 1 mm NaF, and 1 mm phenylmethylsulfonyl fluoride (PMSF)]. The samples were sonicated and then centrifuged at 16 000 ***g*** for 20 min. Proteins were quantified with a Bradford assay kit used according to the manufacturer's protocol (Bio‐Rad, Hercules, CA, USA). Extracts were heated at 95 °C for 5 min, resolved on 10% separating polyacrylamide gel, and transferred to nitrocellulose membranes. Membranes were blocked in Tris‐buffered saline with 0.1% Tween‐20 (TBS‐T) and 5% dry milk (w/v) for 1 h and then washed three times in TBS‐T. Membranes were incubated overnight at 4 °C with primary antibodies and probed with HRP‐conjugated secondary antibodies for 1 h. The blots were visualized using chemiluminescence with the ECL detection system (Amersham International PLC, Buckinghamshire, UK). Western blot densitometry quantification was done using imagej software (version 1.46r; NIH, Bethesda, MD, USA). Protein levels were normalized with the levels of the loading control.

### AMPKα, p38MAPK, metrnl, and TBC1D1 silencing

Transient transfections were performed using Lipofectamine 2000 (Invitrogen, Carlsbad, CA, USA) according to the manufacturer's protocol. Briefly, AMPKα2 siRNA (L‐NM_100623), p38MAPK siRNA (L‐040125‐00), TBC1D1 siRNA (L‐040360‐01) (Dharmacon, Lafayette, CO, USA), metrnl siRNA (L‐059938‐01), and non‐targeting pool siRNA (L‐001810‐10, On‐TARGET plus SMART pool oligonucleotide; Dharmacon) were used. For each experiment, 5 μL of Lipofectamine 2000 was diluted in 95 μL of reduced‐serum medium (Opti‐MEM; Invitrogen) and then mixed with siRNA. The mixtures were incubated for 15 min and then added dropwise to culture wells containing 800 μL of Opti‐MEM to achieve a final siRNA concentration of 50 nm.

### Reverse transcription polymerase chain reaction and real‐time PCR

Reverse transcription polymerase chain reaction (RT‐PCR) was performed at 55 °C for 20 min using a Thermoscript II one‐step RT‐PCR Kit (Invitrogen). cDNA amplification was carried out using a Gene Amp System 9700 thermocycler (Applied Biosystems, Warrington, UK). The reverse transcriptase was heat‐inactivated in the first step of the PCR (95 °C for 10 min). The following primers were used for amplification: metrnl, sense 5′‐AAGCCTTTCAGGGACTCCTC‐3′ and antisense 5′‐CCCTGGTCGTACTCCACACT‐3′; β‐actin, sense 5′‐ATTTGGTCGTATTGGGCGCCTGGTCACC‐3′ and antisense 5′‐GAAGATGGTGATGGGATTTC‐3′; GLUT4 for real‐time PCR, sense 5′‐AGCTGGTGTGGTCAATACGG‐3′ and antisense 5′‐AACAGATGGAGTGTCCGTCG‐3′; GLUT4 for ChIP, sense 5′‐CTTCGACCTTTCAGGGGGAC‐3′ and antisense 5′‐GAACAAAAGGCTCTTCCCGC‐3′. The amplification steps were as follows: 32 cycles of 95 °C for 15 s, 58 °C (β‐actin) or 55 °C (GLUT4 and metrnl) for 30 s, and 72 °C for 30 s, followed by 10 min at 72 °C. After each reaction, 10 µL was analyzed by agarose gel electrophoresis. For real‐time PCR, the relative amount of the target genes was determined by measuring the cycle threshold values of the target genes and β‐actin. The relative amount of the target genes was normalized against β‐actin, the internal control in the same sample, and described as the ratio of each target gene/β‐actin.

### Myc‐GLUT4 immunodetection

The cell surface expression of Myc‐GLUT4 was quantified using a previously described antibody‐coupled colorimetric absorbance assay [17]. Following stimulation, differentiated L6 myotubes stably expressing Myc‐GLUT4 were incubated with a polyclonal anti‐Myc antibody (1 : 1000) for 60 min and then incubated with an HRP‐conjugated goat anti‐rabbit IgG (1 : 1000) for 1 h. Cells were washed six times with PBS and incubated in 1 mL of *o*‐phenylenediamine dihydrochloride (OPD) reagent (0.4 mg·mL^−1^) for 30 min. The absorbance of the supernatant was measured at 492 nm.

### Glucose uptake

Differentiated C2C12 myotubes were washed twice with PBS and then starved in serum‐free low‐glucose DMEM for 3 h. The cells were next incubated with KRB [20 mm HEPES (pH 7.4), 130 mm NaCl, 1.4 mm KCl, 1 mm CaCl_2_, 1.2 mm MgSO_4_, and 1.2 mm KH_2_PO_4_] and then incubated with test compounds in the same buffer at 37 °C. The uptake assay was initiated by adding 2‐deoxy‐d‐(H^3^)‐glucose (2‐DG) to each well and incubating at 37 °C for 15 min. The reaction was terminated by washing with ice‐cold PBS. Cells were lysed in 10% SDS. An aliquot of the cell lysate was removed for protein quantitation by the Bradford assay method. The uptake of [H^3^]‐2‐deoxyglucose was determined (in triplicate) by scintillation counting.

### Immunoprecipitation

Cellular proteins (1 mg) were mixed with 1 μg of anti‐14‐3‐3 (rabbit monoclonal antibody) or anti‐IgG (normal rabbit antibody) and incubated at 4 °C for 24 h. Immune complexes were captured using protein A‐Sepharose beads (Amersham, Uppsala, Sweden) for a further 3 h. The precipitated immune complexes were washed three times with a wash buffer [25 mm HEPES, 5 mm EDTA, 1% Triton X‐100, 50 mm NaF, 150 mm NaCl, 10 mm PMSF, 1 μm leupeptin, 1 μm pepstatin, and 1 μm aprotinin (pH 7.2)]. The washed samples were resuspended in SDS sample buffer [125 mm Tris/HCl (pH 6.8), 20% (v/v) glycerol, 4% (w/v) SDS, 100 mm dithiothreitol, and 0.1% (w/v) bromophenol blue] and heated at 100 °C for 5 min.

### Isolation of the plasma membrane fraction

C2C12 mouse myoblast cells (2 × 10^7^) were plated in treated 10‐cm cell culture dishes, the growth medium was changed to Opti‐MEM for 6 h, and then, the cells were treated with metrnl (100 ng·mL^−1^) for 3 h or 100 nm insulin for 30 min. The supernatants were removed, cells were washed three times with ice‐cold PBS, and the plasma membrane was extracted and purified using a plasma membrane protein extraction kit (ab65400; Abcam, Boston, MA, USA) according to the manufacturer's instructions.

### Chromatin immunoprecipitation assay

A ChIP assay was performed using a kit (Cell Signaling Technology, MA, USA) according to the manufacturer's instructions. C2C12 myoblasts were treated with metrnl, and then, DNA–protein complexes were cross‐linked using 1% formaldehyde for 15 min and quenched using 125 mmol·L^−1^ glycine. The cross‐linked chromatin samples were isolated from the cell lysates by nuclease digestion. HDAC5 was immunoprecipitated using an HDAC5 antibody (Novus Biologicals, Littleton, CO, USA) or an unrelated control antibody (normal rabbit IgG) and then DNA was extracted. For quantitative PCR, ChIP DNA was amplified using the following primers for the GLUT4 promoter containing the MEF2 binding site: forward 5′‐CTT CGA CCT TTC AGG GGG AC‐3′ and reverse 5′‐GAA CAA AAG GCT CTT CCC GC‐3′. Each reaction used Power SYBR green PCR Master Mix (Applied Biosystems, Foster City, CA, USA). Values represent enrichment over the IgG negative control using the threshold cycle ($2^{-\Delta \Delta C_{T}}$) method.

### Immunocytochemistry

Cells were fixed with 4% paraformaldehyde (PFA)/PBS at room temperature for 15 min. After blocking with 3% bovine serum albumin (BSA) at room temperature for 30 min, the fixed cells were incubated with primary anti‐p‐HDAC5 (1 : 500, gtx50238; Gene Tex, Irvine, CA, USA), primary anti‐p‐TBC1D1 (1 : 500, 07‐2268; Merck Milipore, Damstadt, Germany), or primary anti‐14‐3‐3 (1 : 500, ab6081; Abcam) antibodies in primary antibody diluent (PBS, 3% BSA, and 0.1% Triton X‐100) at 4 °C overnight. Cells were then washed with PBS, probed with a goat anti‐rabbit Cy3 (red) or goat anti‐mouse 488 (green) secondary antibody (Molecular Probes, Eugene, OR, USA) and washed three times with PBS at room temperature for 10 min. Images were obtained using a Zeiss confocal microscope (LSM700).

### Plasmid construction of GFP‐GLUT4 and GST‐metrnl

A mouse myc‐DDK‐tagged GLUT4 ORF clone (MR_208202) and a mouse metrnl ORT clone (MR_14497) were purchased from Origene Technologies, Inc. (Rockville, MD, USA). Plasmid DNA from those clones was amplified by PCR using the following primers: GLUT4, forward 5′‐CGCGGGCCCGGGATCC ATG CCT TCG GGT TTC CAG CAG‐3′ and reverse 5′‐G AGC TCG CAA ACA GAG CTG AAC TAG‐3; metrnl, forward 5′‐GGT TCC GCG TGG ATC CCA GTA CTC CAG CGA CCT G‐3′ and reverse 5′‐GAT GCG GCC GCT CGA GCT CCA TAT TGA TTT CAC A‐3′. The BamH1‐ and Sac1‐digested products of GLUT4 and BamH1‐ and Xho1‐digested products of metrnl were ligated into linearized pEGFP‐C1 (Clontech, Palo Alto, CA, USA) and pGEX4‐1 vectors (GE Healthcare, Boston, MA, USA), respectively. All constructs were verified by direct sequencing. Nuclei were stained with Hoechst 33342 dye for 30 min at 25 °C. Confocal images were obtained using a Zeiss confocal microscope (LSM700) and analyzed using the zeiss lsm image browser software.

### Confocal microscopy

C2C12 cells expressing GFP‐GLUT4 were fixed with 4% PFA/PBS at room temperature for 15 min. After blocking with 3% BSA at room temperature for 30 min, the nuclei were stained with Hoechst 33342 dye for 30 min at 25 °C. Confocal images were obtained using a Zeiss confocal microscope (LSM700) and analyzed using the zeiss lsm image browser software.

### Purification of GST‐metrnl and GST proteins and administration in mice

The GST and GST‐metrnl fusion proteins were expressed in *E. coli* and purified using glutathione agarose beads (GE Healthcare) according to the manufacturer's instructions. The purity and integrity of the fusion proteins were analyzed by SDS/PAGE and Coomassie blue staining. Diabetic (db/db) and HFD mice were given 3 mg·kg^−1^ of recombinant GST or GST‐metrnl by intraperitoneal injection for 4 weeks.

### Mouse metrnl sandwich ELISA

Immediately after exercise, blood was collected from control and exercised mice, and samples were centrifuged at 3000 r.p.m. for 10 min at 4 °C. The serum metrnl concentration was measured using a mouse metrnl ELISA kit (Cusabio) according to the manufacturer's instructions.

### *Ex vivo* glucose uptake

Primary myoblasts were obtained from the forelimbs and hind limbs of 5‐day‐old littermate pups (*n* = 3–4). Dissected and minced muscle was enzymatically disaggregated at 37 °C in 4 mL PBS containing 1.5 U·mL^−1^ dispase II and 1.4 U·mL^−1^ collagenase D (Roche, Penzberg, Germany). Samples were sheared by mixing with a 10‐mL pipette every 5 min for 20 min. Cells were filtered through a 70‐µm mesh and collected by pelleting at 1230 ***g*** for 5 min. The cell pellet was dissociated in 10 mL F10 medium (Invitorgen, Valencia, CA, USA) supplemented with 10 ng·mL^−1^ basic fibroblast growth factor (Pepero Tech, Rocky Hill, NY, USA) and 10% Cosmic calf serum (Hyclone, Logan, UT, USA). Cells were pre‐plated twice onto non‐collagen‐coated plates for 1 h to deplete the fibroblasts. After the cells reached confluence, differentiation was induced by incubation in DMEM supplemented with 2% FBS for 2 days. Cells were washed twice with PBS and then starved in serum‐free, low‐glucose DMEM for 3 h. Next, the cells were incubated with KRB and incubated with the indicated compounds at 37 °C. The uptake assay was initiated by adding 2‐DG to each well and incubating at 37 °C for 15 min. The reaction was terminated by washing with ice‐cold PBS. The cells were lysed in 10% SDS and mixed with a scintillation cocktail to measure radioactivity. This experiment was approved by the Korea University Institutional Animal Care and Use Committee and was performed in accordance with its guidelines and regulations. The glucose uptake experiment reported in Fig. 9C was conducted as previously described [51].

### Electrical pulse stimulation of muscle cells

Electrical pulse stimulation experiments were performed according to the method of Lambernd *et al*. [52]. In short, myotubes were starved overnight in serum‐free DMEM to exclude the effects of the many undefined factors in FBS. The starvation medium was refreshed directly before stimulation to minimize the effects of factors potentially secreted during the overnight starvation. EPS was applied to C2C12 myotubes cultured under high‐density micro‐mass conditions (2 × 10^5^ cells·mL^−1^) using either an acute protocol (pulse trains of bipolar pulses at 1 Hz for 2 ms every 5th second, 25 V, for 1–6 h) or a chronic protocol (single, bipolar pulses of 2 ms, with 25 V and 1 Hz continuously for the last 12, 24, or 36 h of the differentiation period) in a C‐dish with carbon electrodes combined with a pulse generator (C‐Pace 100; Ion Optix, Milton, MA, USA). At the indicated time points, cells were harvested in Trizol (Invitrogen) for PCR analysis or in lysis buffer for western blotting.

### Treadmill running procedure

Eight‐week‐old, specific‐pathogen‐free, male BALB/C mice were maintained according to Korea University College of Medicine research requirements. All procedures were approved by the Committee on Animal Research. The animals were fed chow and water *ad libitum* and acclimatized to a 12‐h light cycle (lights on between 0600 and 1800 h) for 1 week before experimental manipulation. Mice were divided into two groups, one of which received treadmill training for three weeks. The forced exercise was performed at a velocity of 10 m·min^−1^ for 60 min and was administered 5 days·week^−1^. After the final exercise session on day 21, the animals were anesthetized with zoletil (Virbac Laboratories, Carros, France) by intraperitoneal injection. Blood samples were harvested by cardiac puncture into tubes containing heparin solution and then centrifuged at 2000 ***g*** for 10 min to obtain plasma for ELISA testing.

### Animals and experimental design

For the diet‐induced obesity experiments, 50 specific‐pathogen‐free C57BL/6N male mice (7 weeks old, 22–24 g) were prepared and fed an HFD or NCD. Seven‐week‐old male db/m and db/db mice (C57BL KSJ M^+^/lepR^−/−^) were supplied by Central Lab Animal, Inc. (Seoul, Korea) for the diabetic mouse experiments. All procedures were approved by the Committee on Animal Research. For the obese mice, 8‐week‐old C57BL/6N males were divided randomly into four groups of ten animals. The first and second groups were then fed an HFD and injected with recombinant GST or recombinant metrnl‐GST for 8 weeks. The third and fourth groups were fed an NCD and injected with GST or recombinant metrnl‐GST for 8 weeks. For the diabetic mice, eight‐week‐old db/db males were divided randomly into two groups of 10 animals. The db/m group was the positive control. The first group of db/db mice was injected with recombinant GST, and the second group was injected with recombinant metrnl‐GST three times per week for 8 weeks. After the final treatment with recombinant protein, GTTs were performed. All experimental animals fasted for ~ 16 h before 20% glucose (2 g·kg^−1^) was injected intraperitoneally. Blood glucose levels were measured before the injection and 15, 30, 60, and 120 min after injection. Blood glucose concentrations were measured using an Accu‐Check glucometer (Roche). The experiments with the AMPK β1β2 MKO mice were performed by our collaborators in Melbourne. All animal procedures were approved by the St. Vincent's Hospital Animal Ethics Committee or the Ethics Committee of the Life Sciences Sector, Université Catholique de Louvain (Male Wistar mouse). Information about the AMPK β1β2M‐KO mice was published previously [31].

### Blood and tissue samples

To rule out temporary training effects in the mice, tissue sampling was conducted 6 h after the completion of the last exercise. After complete anesthesia (ethyl ether), blood samples (1 mL) were obtained from the abdominal vena cava via syringes. Plasma was collected using centrifugation of heparinized blood at 16 000 ***g*** for 15 min. After blood sampling, abdominal visceral fat and right leg muscle tissue were excised and weighed. The tissues and plasma were then stored at −80 °C until analysis.

### Statistical analysis

All data are presented as the mean ± standard error of the mean (SEM). Differences between treatment groups were tested using one‐way ANOVA with Bonferroni's *post hoc* test. Statistical difference between the two groups was determined by Student's *t*‐test. Differences with *P* < 0.05 were considered statistically significant. Statistical analyses were performed using sigma plot 12.0 (Systat Software Inc., London, UK).

## Conflict of interest

The authors declare no conflict of interest.

## Author contributions

K‐HS and LJO conceived the study and designed the experiments. WSB, MJK, JM, JAH, M‐JS, HJL, J‐SL, C‐GS, KMK, and SL performed the cell culture and western blot experiments. SKH and KTW carried out Chip analysis. E‐SL, HMK, and CHC performed animal experiments. KRWN, NXYL, JSO, SG, LM‐S, and BEK performed AMPK β1β2 MKO mice experiments. K‐HS, LJO, NXYL, and KRWN analyzed the data and wrote the manuscript.
